# Nutrition knowledge, attitudes toward physical activity and malnutrition as predictors of social appearance anxiety: a structural equation modeling approach

**DOI:** 10.3389/fnut.2025.1668374

**Published:** 2025-10-08

**Authors:** Fatih Harun Turhan, Uğur İnce, Mehmet Özkeskin, Numan Bahadır Kayışoğlu, Mehmet Ali Öztürk, Nur Elvan Koç Doğan, Musa Kızıltaş, Berkay Karagöz, İsa Doğan

**Affiliations:** ^1^Hasan Dogan Faculty of Sport Sciences, Karabük University, Karabük, Türkiye; ^2^Mehmet Arabacı School of Physicial Education and Sport, Haran University, Şanlıurfa, Türkiye; ^3^Faculty of Sport Sciences, Dumlupinar University, Kütahya, Türkiye

**Keywords:** social appearance anxiety, nutrition knowledge, physical activity attitudes, malnutrition, structural equation modeling

## Abstract

**Background:**

Social appearance anxiety (SAA) has emerged as a critical psychosocial concern among young adults, influenced by various behavioral and cognitive factors. Despite growing recognition of the role of health literacy, limited research has examined how nutrition knowledge may influence SAA through indirect pathways.

**Objective:**

This study aimed to examine the predictive role of nutrition knowledge on social appearance anxiety, with a particular focus on the mediating effects of physical activity attitudes and malnutrition.

**Methods:**

A cross-sectional design with a convenience sample of 338 university-level athletes (Mage = 22.04, SD = 3.46) was employed. Participants completed validated measures of nutrition knowledge, attitudes toward physical activity, malnutrition, and SAA. Structural Equation Modeling (SEM) was used to analyze direct and indirect pathways. Model fit indices and bootstrapping methods were used to test mediation effects.

**Results:**

Nutrition knowledge was negatively associated with SAA (β = −0.19, *p* < 0.001). However, this direct relationship lost significance when physical activity attitudes and malnutrition were introduced as parallel mediators. Mediation analysis revealed significant indirect effects of nutrition knowledge on SAA through both physical activity attitudes (β = −0.30, *p* < 0.001) and malnutrition (β = −0.42, *p* < 0.001), supporting a full mediation model. The final model explained 27% of the variance in social appearance anxiety and demonstrated satisfactory fit indices (e.g., RMSEA = 0.065; CFI = 0.93).

**Conclusion:**

The findings underscore the importance of targeting both behavioral (physical activity) and physiological (malnutrition) mediators when addressing the impact of nutrition knowledge on social appearance anxiety. Interventions designed to reduce SAA should incorporate components that enhance nutritional literacy and promote positive lifestyle behaviors to improve mental and physical well-being among young adults.

## 1 Introduction

There is an increasing acknowledgment of nutrition knowledge as a fundamental determinant shaping individuals’ dietary quality and general lifestyle behaviors (1–4). A substantial body of recent literature has demonstrated that the implications of nutrition knowledge extend beyond physical health, revealing indirect effects on psychosocial outcomes, such as social appearance anxiety (4–10). Furthermore, social appearance anxiety has emerged as a prevalent issue within contemporary societies, particularly affecting young adults and precipitating negative outcomes on body image and self-esteem (11, 12). According to the findings of Brosof and Levinson (5), existing evidence suggests that nutrition knowledge exerts an indirect influence on anxiety levels.

In this context, positive attitudes toward physical activity have been proposed as a complementary factor in elucidating the relationship between nutrition knowledge and social appearance anxiety (13). Indeed, individuals with elevated nutrition knowledge often exhibit more positive dispositions toward physical activity, yielding beneficial effects on both body composition and perceived body image (14, 15).

Conversely, maladaptive dietary behaviors, notably the excessive consumption of high-calorie, nutrient-poor foods, have been associated with negative body alterations that exacerbate social appearance anxiety (16). Empirical studies, particularly those involving young adults, have consistently confirmed the correlation between dietary habits and social appearance anxiety (4–9). Furthermore, the extant literature has reported associations between social appearance anxiety and perfectionism, as well as eating disorders (5). Elevated levels of social appearance anxiety have been identified in individuals with eating disorders (6), while dieting motivations have been linked to social pressures and appearance-related anxieties (4). Further evidence suggests potential associations between nutrition knowledge and anxiety (9), thus indicating that enhanced nutritional literacy may concomitantly elevate quality of life and mitigate anxiety (17).

Despite these insights, current literature appears limited in explicitly addressing the direct influence of nutrition knowledge on social appearance anxiety and inadequately examines the mediating roles of physical activity, attitudes and maladaptive dietary practices. The present study has been designed to make a novel contribution to the field by investigating the relationship between nutrition knowledge and social appearance anxiety. The investigation will explore the mediating mechanisms of physical activity, attitudes and maladaptive dietary habits. Moreover, the objective is to furnish findings that may assist individuals experiencing social appearance anxiety in the regulation of dietary behaviors, the comprehension of the significance of attitudes toward physical activity, and thereby the acquisition of a more profound sense of self-awareness.

Inadequate nutrition has been demonstrated to be closely linked not only to physiological health outcomes, but also to individuals’ body image and psychosocial well-being. Within the context of social cognitive theory and health behavior models (e.g., the Health Belief Model), it is emphasized that dietary behaviors play a decisive role in individuals’ self-efficacy, perceived body appearance, and need for social approval (18, 19). Excessive consumption of high-calorie, low-nutrient foods has been demonstrated to have a detrimental effect on an individual’s physical health, often resulting in weight gain and undesirable body changes. These changes can, in turn, lead to an increased sense of social appearance anxiety. This has been identified in the literature as a significant predictor of body dissatisfaction, paralleling eating disorders and perfectionism (6, 16). Consequently, inadequate nutrition has been demonstrated to engender adverse health consequences, in addition to giving rise to concerns regarding an individual’s social perception. Consequently, the incorporation of malnutrition as a mediating mechanism within the model, thereby elucidating the relationship between nutritional knowledge and social appearance anxiety, is theoretically congruent.

The study is structured around the following hypotheses, explicitly formulated to elucidate the mediating roles of physical activity attitudes and maladaptive dietary practices in the association between nutrition knowledge and social appearance anxiety:

The present study hypothesizes that nutrition knowledge significantly predicts social appearance anxiety.

As demonstrated in the extant literature, nutritional knowledge has been shown to have a significant predictive capacity with regard to physical activity, attitude and malnutrition.

The relationship between nutritional knowledge and social appearance anxiety is mediated by physical activity, attitude and malnutrition, which act in a parallel manner.

## 2 Materials and methods

### 2.1 Research pattern

The present study employed the convenience sampling method to collect data, and structural equation modeling (SEM) was utilized (20). Nutrition knowledge was defined as an exogenous variable in the structural equation model, while social appearance anxiety was defined as an endogenous variable. It was hypothesized that attitudes toward physical activity and malnutrition would not be independent variables, but rather would function as mediators of the relationship between nutrition knowledge and social appearance anxiety. In the present study, these variables were modeled as parallel mediators.

### 2.2 Study group/population sample

The study population comprised students enrolled in a faculty of sport sciences who had been actively participating in licensed sports for a minimum of 5 years. Consequently, the generalizability of the findings is constrained to this particular sample. The study excluded young adults who were not athletes, students studying at other faculties, or individuals who had been involved in sports for a shorter period of time. Moreover, individuals diagnosed with psychological disorders were not included in the study. This limitation resulted in an inability to examine the potential effects of psychiatric disorders or mental health problems on social appearance anxiety. The findings demonstrate that the observed relationships are only indicative of concurrent relationships between relevant variables. The data were collected via self-report questionnaires. It is important to note that this method may not fully reflect the participants’ actual attitudes and behaviors due to social desirability bias and respondent bias. The study sample was recruited exclusively from select universities in Turkey. Consequently, studies undertaken in disparate cultural, socioeconomic, or geographical contexts may yield divergent results. A total of 338 athletes voluntarily participated in this study. The mean age of the participants was 22.04 years (SD = 3.46), ranging from 18 to 45 years. The demographic composition of the sample was as follows: 62.4% of the participants were female and 37.6% were male. The following procedure is to be observed:

All stages of the study were conducted in accordance with the Declaration of Helsinki. The data for this study was collected using an online survey. The response time to the questionnaire was reported as 10 min. In the first instance, participants were furnished with a concise explanation of the objective of the study. This was done on the first page of the online questionnaire. Participants were required to be over the age of 18 years in order to be included in the study. Prior to commencing the study, written consent was obtained from all participants. The anonymity and confidentiality of responses was ensured. The scales were administered to students who voluntarily agreed to participate in the study.

The study did not include students under the age of 18. The rationale behind the decision to establish an age criterion of 18 for the participants is outlined below. Initially, the age criterion for the adapted questionnaires was set at 18 years and above. Furthermore, one of the primary criteria was the selection of university students with regard to accessibility to the participants (*N* = 338). However, it is evident that the theoretical substructure of the study’s variables centers on the mental health of individuals aged 18 and over.

### 2.3 Data collection tools

#### 2.3.1 Social appearance anxiety scale-(SAAS)

The Social Appearance Anxiety Scale (SAAS), developed by Hart et al. (21) and adapted into Turkish by Doğan (22), is a self-report scale used to measure individuals’ emotional, cognitive and behavioral concerns about their appearance. The SAAS is a 16-item, 5-point Likert-type scale. For instance, an individual might articulate their sentiments as such: “I experience a state of anxiousness when I am the subject of others’ direct gaze.” The Social Security Inventory (SAAS) utilizes a 5-point Likert-type response key, ranging from (1) Not Appropriate at All to (5) Completely Appropriate. Item 1 of the scale is reverse-coded. The attainment of elevated scores on the SAAS, a tool designed to measure social appearance anxiety in a unidimensional manner, is indicative of a high level of appearance anxiety. In the present study, the internal consistency coefficient was determined to be 0.961.

#### 2.3.2 Attitude scale toward healthy nutrition (ASTHN)

The scale developed by Tekkurşun Demir and Cicioğlu (23) was created for the purpose of evaluating attitudes toward healthy eating. The scale under consideration consists of 21 items and four subscales, and each item is rated on a 5-point Likert-type scale ranging from 1 (strongly disagree) to 5 (strongly agree). A sample item is “I know what healthy foods are.” A higher score on the scale indicates a greater level of meaning is attributed to nutritional information. In this study, the internal consistency coefficient was calculated to be 0.958.

#### 2.3.3 Attitudes toward physical activity (ATPA)

The scale developed by Savaş and Çelik-Kayapınar (24) was utilized as a tool to measure individuals’ attitudes toward physical activity. The scale under consideration consists of 25 items and two subscales, and each item is rated on a 5-point Likert-type scale ranging from 1 (strongly disagree) to 5 (strongly agree). For instance, the following statement is provided: “Physical activity strengthens me.” A total score on the scale ranging from 25 to 57 indicates a low attitude; a score between 58 and 91 indicates a moderate attitude; and a score between 92 and 125 indicates a high attitude. In the present study, the internal consistency coefficient was calculated as 0.837.

#### 2.3.4 Data analysis

The Structural Equation Modeling (SEM) data analysis was conducted using AMOS 24 and SPSS 25 package programs. The SEM can be regarded as a multivariate statistic, the basis of which is founded upon certain fundamental structures, including, but not limited to, normality, extreme value, linearity, multicollinearity, and magnitudes (25). In consideration of the path coefficients and model fit as reported in this study, a minimum range of *N* ≈ 180–250 is deemed adequate to detect parallel mediation effects with over 80% power and to assess fit based on RMSEA (26, 27). Consequently, the current *N* = 338 is more than sufficient in terms of power and stability.

Moreover, In this study, nutritional knowledge and malnutrition variables were measured using the sub-dimensions of the Attitudes Toward Healthy Nutrition Scale developed by Tekkurşun Demir and Cicioğlu (23). Of the four sub-dimensions of the scale, the “Nutrition Knowledge” sub-dimension was defined as an exogenous variable in the study, while the “Malnutrition” sub-dimension was modeled as a mediator variable. Consequently, the nutritional knowledge and malnutrition variables employed in the study were directly operationalized with these scale sub-dimensions and incorporated into the analyses. The utilization of these sub-dimensions of the scale guarantees conceptual consistency and furnishes a valid and reliable basis for measuring the variables.

The validity of these assumptions for SEM has been examined, and the results are given below. The findings of the correlation analysis and the assumptions of the structural equation model lend support to the model that emerged among the variables of this study (Table 1). Following the preliminary analyses, CFA was conducted to test the proposed model. According to the results of the analysis, the model fit values of the variables are as follows: CMIN = 871.96; df = 366; CMIN/df = 2.38; RMSEA = 0.064, CFI = 0.93, TLI = 0.92, and IFI = 0.93. Mediation analyses were then tested with a structural equation model. The analyses started by creating the first model for the hypothesis H1. The diagram of the model is shown in (Figure 2).

**TABLE 1:** Descriptive Statistics, Correlations, Linearity, Normality, and Multicollinearity (*N* = 338).

Variable	Mean	SD	Skew.	Kurt.	1.	2.	3.	4.	VIF	Tolerance
1. Social appearance anxiety	2.42	0.97	0.34	−0.75	–	−0.19*	−0.36*	−0.44*	–	–
2. Nutrition knowledge	4.08	0.60	−0.47	0.82		–	0.47*	0.24*	1.296	0.77
3. Physical activity attitude	12.96	1.61	−0.55	−0.13			–	0.32*	1.354	0.74
4. Malnutrition	3.42	0.87	−0.25	−0.44				–	1.125	0.89

*P < 0.05

### 2.4 Research ethics

Ethical approval to administer the scales and collect data was granted by the Non-Interventional Research Ethics Committee of Karabuk University (Approval Date: 11/10/2024, Meeting No: 2024/9; E-381436, Decision No: 26). Participation in this study was entirely voluntary.

## 3 Results

The descriptive statistics and correlation matrix for the study variables are presented above. The mean scores indicate moderate levels of social appearance anxiety (*M* = 2.42, SD = 0.97) and malnutrition (*M* = 3.42, SD = 0.87), relatively high nutrition knowledge (*M* = 4.08, SD = 0.60), and physical activity attitude (*M* = 12.96, SD = 1.61). The skewness and kurtosis values for all variables fall within acceptable ranges, suggesting that the data are approximately normally distributed.

A substantial correlation was identified between the variables. The present study examined the relationship between social appearance anxiety and nutrition knowledge, physical activity attitude, and malnutrition. The findings indicated a negative correlation between social appearance anxiety and nutrition knowledge (*r* = −0.19, *p* < 0.05), physical activity attitude (*r* = −0.36, *p* < 0.05), and malnutrition (*r* = −0.44, *p* < 0.05). These results suggest that individuals with higher social appearance anxiety tend to have lower nutrition knowledge, less positive attitudes toward physical activity, and higher levels of malnutrition. Furthermore, an affirmative correlation was identified between nutrition knowledge and physical activity attitude (*r* = 0.47, *p* < 0.05) and malnutrition (*r* = 0.24, *p* < 0.05). Multicollinearity diagnostics indicate that all tolerance values are above 0.70 and VIF values are below the commonly accepted threshold of 5, indicating no concerns regarding multicollinearity among the predictors (Table 1).

**FIGURE 1:**
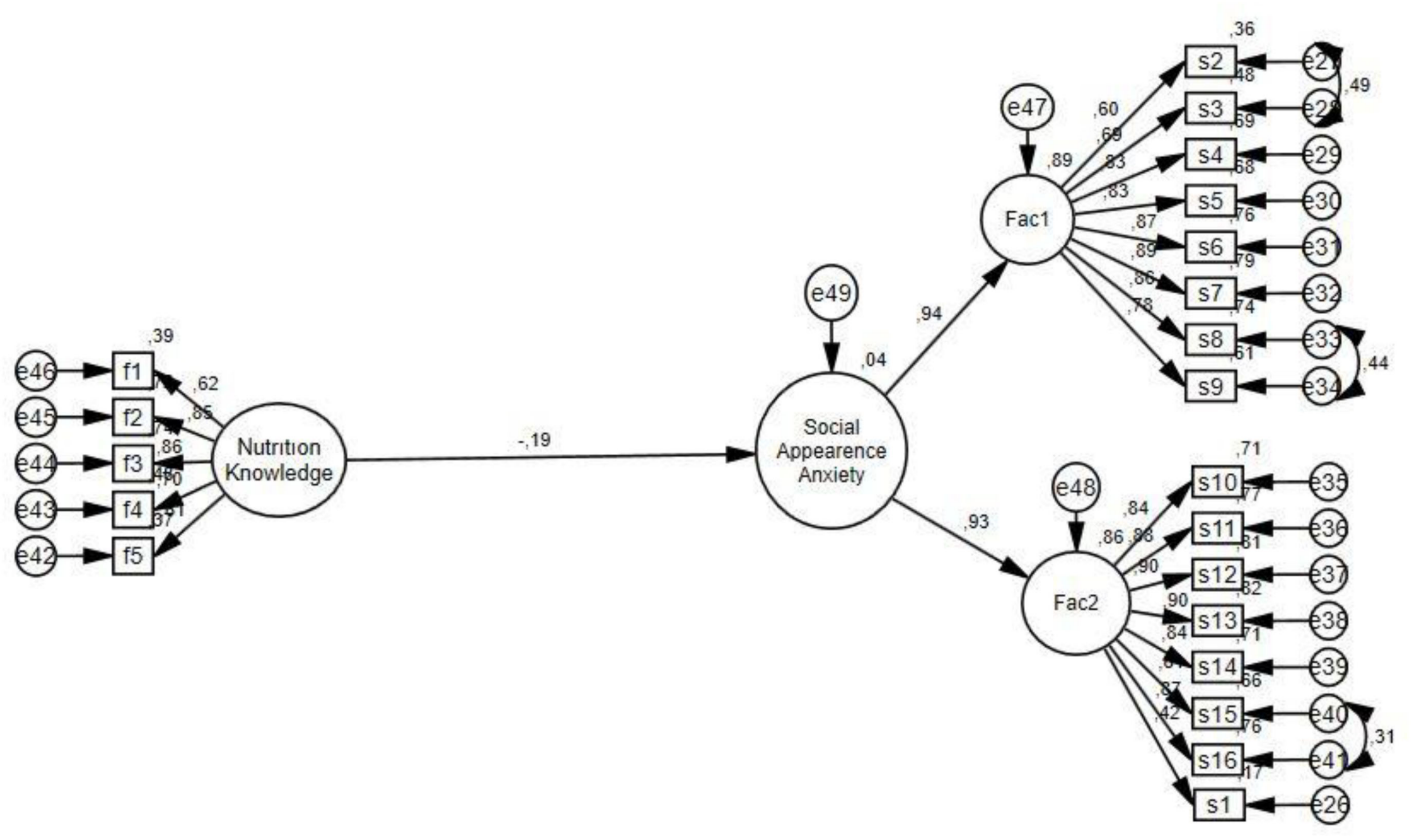
Total effect of nutrition knowledge.

As a result of the covariances, it was seen that nutrition knowledge predicted social appearance anxiety (β = −0.19; *p* < 0.001). The goodness of fit indices resulting from the covariances are as follows: CMIN = 546.49, df = 183, CMIN/df = 2.98, RMSEA = 0.077, CFI = 0.94, TLI = 0.93, and IFI = 0.94. It is evident that the model fit values are deemed to be adequate as in Figure 1.

The regression weights shown in the diagram resulting from including mediator variables in the model were statistically significant (*p* < 0.005). The structural model, including mediator variables, revealed that nutrition knowledge significantly predicted malnutrition (β = 0.29; *p* < 0.001). Similarly, the mediator variable, physical activity attitude, significantly predicted the social appearance anxiety (β = −0.30; *p* < 0.005). It was revealed that nutrition knowledge significantly predicted physical activity attitude (β = 0.52; *p* < 0.001). It was revealed that malnutrition predicted the social appearance anxiety (β = −0.42; *p* < 0.001).

Nutrition knowledge and physical activity attitude explained 27% of the variation, malnutrition explained 8%, and social appearance anxiety explained 27% of the variation (square of multiple correlations).

The total effect of nutrition knowledge on social appearance anxiety was β = −0.19 (*p* < 0.001), the effect obtained without the mediation variable. When the mediation variables were included in the model, the effect was β = 0.08 (*p* > 0.001).

**FIGURE 2:**
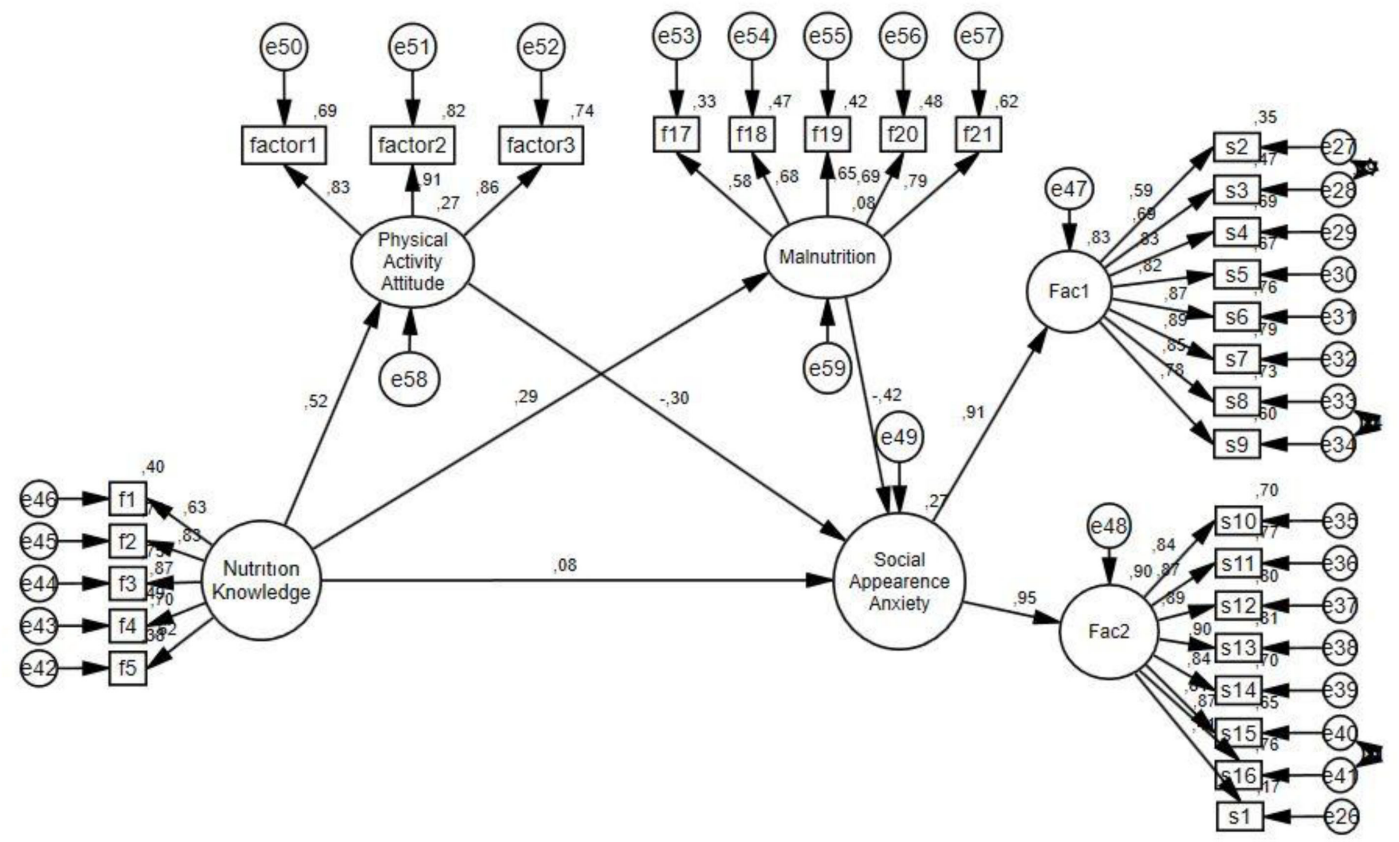
SEM diagram related to the parallel mediation.

The goodness of fit values obtained because of the path analysis in the model are as follows: CMIN = 891.43, df 367, CMIN/df 2.42, RMSEA 0.065, CFI 0.93, TLI = 0.92, and IFI 0.93 (Figure 2).

The bootstrap method was used to check whether physical activity attitude and malnutrition mediate. The mediation model results of the effect of nutrition knowledge physical activity attitude and malnutrition on social appearance anxiety are shown in Table 2.

**TABLE 2:** The result of bootstrapping analyses.

Model pathways	Coefficient	95% CI	
		Lower	Upper
**Direct effects**			
a → b	0.08	−0.06**	0.23**
a → c	0.52	0.40*	0.62*
a → d	0.29	0.16*	0.42*
c → b	0.30	−0.44*	−0.17*
d → b	0.42	−0.55*	−0.27*
**Indirect effects**			
a → b	−0.28	−0.38*	−0.18*

A, nutrition knowledge; b, social appearance anxiety; c, physical activity attitude; d, malnutrition; CI, confidence interval.

*P < 0.05, **p > 0.05 (full medition)

In the parallel mediation analysis (Table 2), it was observed that the direct effect of nutrition knowledge (a) on social appearance anxiety (b) was significant before the inclusion of the mediator variables (physical activity attitude [c] and malnutrition [d]) in the model, but this direct effect lost its significance when the mediator variables were added to the model. This situation shows that the effect of nutrition knowledge on social appearance anxiety is completely mediated by the mediator variables.

As a result of the bootstrap analyses, the total indirect effect of nutrition knowledge on social appearance anxiety was found to be significant (β = −0.28, 95% CI [−0.38, −0.18]). This finding suggests that nutrition knowledge indirectly affects social appearance anxiety through physical activity attitude and malnutrition.

In summary, these results support the full mediation effect; they show that the effect of nutrition knowledge on social appearance anxiety occurs only through the variables of physical activity attitude and malnutrition.

## 4 Discussion

The present study sought to explore the predictive and mediating mechanisms underlying the relationship between nutrition knowledge and social appearance anxiety, with a particular focus on the mediating roles of physical activity attitude and malnutrition. The findings revealed a full mediation model, wherein the impact of nutrition knowledge on social appearance anxiety was completely explained by the parallel mediating effects of physical activity attitude and malnutrition. These results contribute to the growing literature emphasizing the interplay between cognitive, behavioral, and physiological determinants of psychosocial outcomes.

The fact that the sample in this study consisted of students actively participating in university-level sports poses a significant limitation in interpreting the results. Given that athletes frequently expose their bodies in social and performance contexts, the relationships between nutrition knowledge, physical activity attitudes, and social appearance anxiety may be more pronounced or manifest differently than in non-athlete youth. In this context, the full mediation model observed in the study may have been strengthened by the high level of body exposure and performance-oriented lifestyle of the athlete sample.

Consequently, the extrapolation of these findings to non-athlete young adult groups should be approached with a degree of circumspection. Social appearance anxiety in non-athlete youth may be more strongly influenced by various psychosocial factors (e.g., social media use, peer pressure, body dissatisfaction). Consequently, subsequent research conducted with samples of non-athlete youth from diverse disciplines will more clearly demonstrate the extent to which the relationships between nutrition knowledge, physical activity attitudes, and social appearance anxiety are universal or context-specific.

Firstly, the negative indirect association between nutrition knowledge and social appearance anxiety via physical activity attitude aligns with previous research emphasizing the psychological benefits of positive physical activity orientation (7, 28–30). Müftüoğlu and Bayram (31) found that adolescents adhering to healthier dietary habits exhibited both higher physical activity levels and lower levels of social physique anxiety, reinforcing the behavioral mediation mechanism observed in our model (31). Similarly, Alemdağ et al. (32) demonstrated that motivations for engaging in physical activity (e.g., fitness vs. weight loss) significantly affect levels of social appearance anxiety, suggesting that internalized health behaviors can modulate appearance-related concerns (32).

Recent studies on the concept of “Mental Toughness” have demonstrated that psychological resilience acquired through sports directly impacts individuals’ mental health and resilience. For instance, Akoğlu et al. (33) reported that mental toughness levels have direct and significant effects on psychological resilience and mental well-being in both deaf and hard-of-hearing athletes. This finding is consistent with the mediating role of physical activity attitudes in reducing social appearance anxiety in our model, as it supports the positive impact of sports participation and attitudes on psychological outcomes.

Secondly, the mediating role of malnutrition in the nutrition knowledge–appearance anxiety link is a valuable addition to the literature. While most studies emphasize the direct consequences of poor nutrition on health outcomes, recent work by Çomak and Arslan (34) revealed that social appearance anxiety influences food preferences and eating behaviors, suggesting a bidirectional dynamic between appearance concerns and nutritional status (34). Our results expand on this by showing how better nutritional literacy may indirectly improve psychological well-being by reducing malnutrition, thus lowering anxiety related to social appearance.

Furthermore, The findings of this study indicate a positive correlation between nutritional knowledge and malnutrition (*r* ≈ 0.24). The primary rationale for the apparent conceptual incongruity of this finding is that elevated scores on the “Undernutrition” subscale of the Attitudes Toward Healthy Eating Scale are indicative of individuals’ unhealthy eating tendencies. Consequently, individuals with a high level of nutritional knowledge are hypothesized to have lower malnutrition scores. However, the positive correlation found in this study may point to the “knowledge-behavior gap” phenomenon, which has been previously discussed in the literature (3, 9). In summary, despite possessing accurate nutritional knowledge, individuals may persist in maintaining unhealthy eating habits as a result of behavioral, environmental or sociocultural factors. For instance, responses such as “I prefer to consume ready-made foods” or “I enjoy consuming high-calorie foods” are indicative of a tendency toward the malnutrition subscale of the scale. The items under consideration are indicative of the practical behaviors exhibited by individuals. Consequently, this finding indicates that a proportion of sports science students, despite possessing a high level of knowledge, may nevertheless exhibit unhealthy eating behaviors, attributable to lifestyle choices or environmental factors. This finding suggests that interventions which focus solely on increasing individuals’ knowledge levels will not be sufficient; behavioral and environmental factors must also be addressed.

Moreover, the complete mediation effect observed in our study contrasts with findings from Koohi and Abbaszadeh (35), who reported a significant direct association between nutrition knowledge and broader dimensions of social health, including social acceptance and integration (35). The divergence may stem from differences in conceptual focus: while Koohi et al. addressed general social functioning, our model specifically targeted appearance-related anxiety, which is more proximal to body image and health behaviors.

The findings of the present study demonstrate a marked discrepancy when compared to those of studies in the literature reporting direct effects of nutrition knowledge on mental health indicators [e.g., (9, 17)]. The extant literature suggests a direct correlation between nutrition literacy and anxiety, as well as quality of life. In contrast, the effect of nutrition knowledge on social appearance anxiety in our current study emerged solely through behavioral (physical activity attitudes) and dietary (undernutrition) mediators. This finding provides a distinctive contribution to the literature by demonstrating that knowledge alone does not have a transformative effect on mental health outcomes, but becomes significant when reflected in lifestyle practices.

Moreover, while the present study positions malnutrition as a mediating variable, extant literature demonstrates that social appearance anxiety can also lead to malnutrition [e.g., (34)]. This finding suggests the possibility of a bidirectional relationship between the variables. Consequently, future longitudinal and experimental research should examine the interactions between nutritional knowledge, malnutrition, and social appearance concerns in a more holistic manner. This approach will provide a clearer demonstration of the impact of both knowledge and nutritional practices, particularly among young adults, on psychosocial outcomes.

It is also important to note that Sanlier et al. (36) found a negative relationship between social appearance anxiety and various healthy lifestyle behaviors, such as stress management and interpersonal relationships (36). This supports the broader conclusion that enhancing health knowledge can indirectly alleviate psychosocial stressors, a conclusion echoed in our mediated model.

Taken together, these findings collectively suggest that nutrition knowledge exerts its beneficial effects on psychological outcomes not directly, but through its impact on lifestyle behaviors and physiological status. The model demonstrated acceptable fit indices across all stages of the SEM, supporting the robustness of the hypothesized relationships. Furthermore, the variance explained (27% for social appearance anxiety) indicates a meaningful contribution of the proposed predictors, albeit acknowledging the presence of additional, unmeasured influences that merit future investigation.

## 5 Conclusion

This study provides compelling evidence for a fully mediated relationship between nutrition knowledge and social appearance anxiety, via physical activity attitude and malnutrition. These findings highlight the critical role of behavioral and health-related mediators in translating cognitive awareness into psychosocial outcomes. Interventions aimed at reducing social appearance anxiety should not only disseminate nutritional information but also actively promote healthy attitudes toward physical activity and dietary behavior to achieve optimal psychological benefits.

The resulting model accounted for 27% of the variance in social appearance anxiety. This rate can be interpreted as a medium effect size. Consequently, while the model demonstrates substantial predictive capability concerning social appearance anxiety, a considerable proportion of unexplained variance remains. This finding indicates the necessity for future research to incorporate additional psychosocial variables, such as self-esteem, social comparison tendencies, and body image perception, into the model.

However, a pivotal finding of the study is that the direct impact of nutrition knowledge on social appearance anxiety becomes less pronounced when mediators are incorporated. This finding indicates that while nutritional knowledge *per se* may not be adequate to modify social anxiety, it is indicative of psychosocial outcomes through behavioral (e.g., physical activity, attitudes) and habitual (e.g., undernourishment) factors. In summary, knowledge has a limited effect in reducing social appearance anxiety unless it is translated into behavior and daily life practices. This finding suggests that interventions for nutritional literacy should not be limited to the transfer of theoretical knowledge, but should also focus on establishing healthy lifestyle habits in young adults.

Future research should employ longitudinal designs to establish causality and examine whether these mediating mechanisms operate similarly across demographic subgroups. Moreover, integrating psychosocial and physiological assessments could further elucidate the complex pathways linking health knowledge, behavior, and mental health.

## Data Availability

The datasets presented in this study can be found in online repositories. The names of the repository/repositories and accession number(s) can be found in the article/Supplementary material.
