# Supplementary material for: Nutrition knowledge, attitudes toward physical activity and malnutrition as predictors of social appearance anxiety: a structural equation modeling approach
Source: Front Nutr. 2025 Oct 8;12:1668374. doi: 10.3389/fnut.2025.1668374 (PMC12540114; doi:10.3389/fnut.2025.1668374)
Supplement: Supplementary file 1 [file Data_Sheet_1.zip › Etic Desicion Nutri(1).pdf]

T.C.
KARABÜK ÜNİVERSİTESİ REKTÖRLÜĞÜ
Sosyal ve Beşeri Bilimler Araştırmaları Etik Kurulu

Sayı : E-78977401-050.04-389638 18.11.2024
Konu : Etik Kurul Kararı

Sayın Dr. Öğr.Üyesi Fatih Harun TURHAN

İlgi : 09.10.2024 tarihli ve 380481 sayılı dilekçe.

Sosyal ve Beşeri Bilimler Araştırmaları Etik Kurulunda alınan 15.11.2024 tarih ve 2024/09(20,21,22,23,26) sayılı Kurul Kararı yazımız ekinde sunulmuştur. Gereğini rica ederim.

Prof. Dr. Muhittin KAPANŞAHİN
Kurul Başkanı

Ek:Etik Kurul Kararı (5 Sayfa)

Bu belge, güvenli elektronik imza ile imzalanmıştır.
Belge Doğrulama Kodu: BSA8197DLJ Belge Doğrulama Adresi : https://turkiye.gov.tr/ebd?eK=4043&eD=BSA8197DLJ&eS=389638
Adres: Karabük Üniversitesi Merkez Kampüsü Kılavuzlar Mahallesi 413. Sokak No: 10 Merkez/Karabük
Telefon: 444 0478
e-Posta: iletisim@karabuk.edu.tr İnternet Adresi: http://www.karabuk.edu.tr
Kep Adresi: karabukuniversitesi@hs01.kep.tr
Bilgi için: Dilek ERHİN
Unvanı: Bilgisayar İşletmeni
19043

T.C.

KARABÜK ÜNİVERSİTESİ

Sosyal ve Beşerî Bilimler Araştırmaları Etik Kurulu

TOPLANTI TARİHİ: 15.10.2024
TOPLANTI NO : 2024/09

Karabük Üniversitesi Sosyal ve Beşerî Bilimler Araştırmaları Etik Kurulu toplanmış ve aşağıdaki kararı almıştır.

Karar 26:

11.10.2024 tarih ve E. 381436 sayılı Dr. Öğr. Üyesi Fatih Harun TURHAN' ın Etik Kurul form ve ekleri görüşüldü.

Karabük Üniversitesi öğretim üyelerinden Dr. Öğr. Üyesi Fatih Harun TURHAN tarafından yürütülen **"Sağlıklı Beslenme ile Sosyal Görünüş Kaygısı Arasındaki İlişkide Fiziksel Aktiviteye Yönelik Tutumun Aracı Rolü"** konulu çalışma kapsamında uygulanmak üzere ekte sunulan çalışmasının etik kurallara uygunluğu oy birliği ile kabul edilmiştir.

ASLI GİBİDİR

Prof. Dr. Muhittin KAPANŞAHİN
Kurul Başkanı
